# Label-free detection of kanamycin based on a G-quadruplex DNA aptamer-based fluorescent intercalator displacement assay

**DOI:** 10.1038/srep08125

**Published:** 2015-01-30

**Authors:** Yun-Peng Xing, Chun Liu, Xiao-Hong Zhou, Han-Chang Shi

**Affiliations:** 1 State Key Joint Laboratory of ESPC, School of Environment, Tsinghua University, Beijing 10084, China; 2 School of Environmental Science and Engineering, Hebei University of Science and Technology, Shijiazhuang 050000, China

## Abstract

This work was the first to report that the kanamycin-binding DNA aptamer (5′-TGG GGG TTG AGG CTA AGC CGA-3′) can form stable parallel G-quadruplex DNA (G4-DNA) structures by themselves and that this phenomenon can be verified by nondenaturing polyacrylamide gel electrophoresis and circular dichroism spectroscopy. Based on these findings, we developed a novel label-free strategy for kanamycin detection based on the G4-DNA aptamer-based fluorescent intercalator displacement assay with thiazole orange (TO) as the fluorescence probe. In the proposed strategy, TO became strongly fluorescent upon binding to kanamycin-binding G4-DNA. However, the addition of kanamycin caused the displacement of TO from the G4-DNA–TO conjugate, thereby resulting in decreased fluorescent signal, which was inversely related to the kanamycin concentration. The detection limit of the proposed assay decreased to 59 nM with a linear working range of 0.1 μM to 20 μM for kanamycin. The cross-reactivity against six other antibiotics was negligible compared with the response to kanamycin. A satisfactory recovery of kanamycin in milk samples ranged from 80.1% to 98.0%, confirming the potential of this bioassay in the measurement of kanamycin in various applications. Our results also served as a good reference for developing similar fluorescent G4-DNA-based bioassays in the future.

Antibiotics are extensively used to kill or inhibit microorganisms, but their abuse results in various side effects in humans and in the appearance of “superbacteria” with tolerance to antibiotics^1^,^2^,^3^. Therefore, ensuring the use of only suitable amounts of antibiotics is critical. Determining whether food products contain residual antibiotics exceeding the maximum residue limits before being sold and distributed in the market is also important^2^,^4^. As an aminoglycoside antibiotic, kanamycin is widely used as a broad-spectrum antibiotic in veterinary medicine and as a second-line antibiotic in the treatment of serious infections^1^,^2^. However, like other antibiotics, kanamycin abuse has been reported to cause serious side effects, including ototoxicity and nephrotoxicity in humans, especially because of its accumulation effect in human bodies^2^,^5^. Therefore, strict maximum residue limits have been mandated for kanamycin residues, for example, 150 μg/kg in milk (approximately equal to 300 nM) and 100 μg/kg in meat as set by the European Medicines uation Agency^6^. Kanamycin detection is expected to be ubiquitous and frequent not only for contaminant warning but also for trace residual quantification in food monitoring. As a consequence, highly sensitive, selective, and cost-effective assay methods for the detection of residual kanamycin are also necessary to develop.

Until now, different analytical methods have been reported for the detection of residual kanamycin, such as high-performance liquid chromatography^7^,^8^, gas chromatography-mass spectrometry^9^, enzyme-linked immunosorbent assay^10^,^11^,^12^, and surface plasmon resonance^13^. However, these methods tend to be tedious and time-consuming, depend on heavy manual labor and need to consume relatively large amounts of reagents. Therefore, efforts have been devoted to simple, cost-effective, and rapid detection of kanamycin, including colorimetric^2^, electrochemical^4^,^14^,^15^,^16^, and optical^3^ biosensing technologies using antibodies^4^,^14^ and oligonucleotides^2^,^3^,^15^,^16^ as sensing elements.

Aptamers are single-stranded oligonucleotides that have been screened through an in vitro selection process called Systematic Evolution of Ligands by Exponential Enrichment (SELEX)^17^,^18^. Aptamers have emerged as strong competitors for antibodies in analysis applications because they have the advantages of simple production, easy storage, good reproducibility, and particularly target versatility (e.g., ranging from small organic molecules to heavy metals, proteins, cells and even intact viral particles)^18^,^19^,^20^. Very recently, Song et al.^2^ reported a kanamycin-binding DNA aptamer (5′-TGG GGG TTG AGG CTA AGC CGA-3′) that was obtained through SELEX. Benefitting from the reported DNA aptamer, a gold nanoparticle (AuNP)-based colorimetric approach^2^ and a turn-on luminescent probe^3^ were developed for the detection of kanamycin. In studies by Zhu et al.^15^ and Sun et al.^16^, a similar DNA sequence (5′-TGG GGG TTG AGG CTA AGC CGA C-3′) containing the same conserved motif (TTGAGG) for kanamycin-binding was used for the construction of aptamer-based kanamycin biosensing technologies. However, there is still dearth of detailed investigations and well-acceptable views about the conformational characterization of DNA aptamers and the binding principles toward kanamycin, which prohibits the direct application of the results acquired in previous studies into the construction of novel DNA aptamer-based kanamycin bioassays.

In this work, for the first time (as far as we know), we experimentally verify that the kanamycin-binding DNA aptamers can form stable G-quadruplex structures by themselves. G-quadruplex DNA (noted hereafter as G4-DNA) are higher-order DNA and RNA structures formed from G-rich sequences that are built around tetrads of hydrogen-bonded guanine bases and stabilized by stacked G-G-G-G tetrads in monovalent cation-containing solution^21^,^22^,^23^,^24^. Indeed, recent investigations have suggested its critical potential in the design of novel biosensing technologies for different analytes^25^,^26^,^27^,^28^. However, fluorescence based bioassays with G4-DNA are poorly developed, and thus it is appealing for us to further study the possibility of labeling G4-DNA with fluorescent dyes to establish a facile detection strategy for kanamycin.

For fluorescent labeling, the nucleic acid dyes play an increasingly important role in the design of DNA aptamer-based bioassays because of the remarkable advantages such as being label-free and cost-effective, and having high sensitivity and selectivity, for example, the well-known organic dye SYBR Green I^29^,^30^,^31^ and the metal complexes of platinum(II) metallointercalators^3^. These dyes have been reported as fluorescence probes for selectively recognizing the conformational change of DNA; unfortunately, their selectivity largely relies on the formation of double-stranded DNA, such as the T-T mismatch induced by mercury ions^29^, the C-C mismatch induced by silver ions^30^, and the hairpin-like structure induced by kanamycin as proposed by Leung et al.^3^ In 2006, a G-quadruplex Fluorescent Intercalator Displacement (G4-FID) assay was first developed by Teulade-Fichou and coauthors^32^ for determining the quadruplex-DNA binding affinity and sequence selectivity of small-molecule ligands. The assay is based on the loss of fluorescence upon displacement of thiazole orange (TO) from quadruplex- and duplex-DNA matrices. Subsequently, studies have proven the stability of TO dye as the quadruplex- and duplex-DNA stain in the G4-FID assays^33^,^34^,^35^,^36^,^37^,^38^ because it is highly fluorescent upon complexation with DNA, whereas it is totally nonfluorescent when free in solution^39^. Although the G4-FID assay has been established for determining the G4-DNA binding affinity and its selectivity for ligands, no studies have reported using the G4-FID assay in analysis application, such as target quantification.

Motivated by the reported studies and our verification that the kanamycin-binding DNA aptamers (5′-TGG GGG TTG AGG CTA AGC CGA-3′) could form stable G-quadruplex structures by themselves, we established a novel label-free strategy for kanamycin detection based on a G4-DNA aptamer-based FID assay with TO as the fluorescence probe. TO becomes strongly fluorescent upon binding to kanamycin-binding G4-DNA. However, the addition of kanamycin would cause the displacement of TO from the G4-DNA–TO conjugate, thereby resulting in a decrease in the fluorescent signal, which is inversely related with the kanamycin concentration. Based on the proposed strategy, the bioassay is optimized and fully validated in terms of linearity, accuracy, precision, recovery, and specificity.

## Results

### Characterization of kanamycin-binding oligonucleotide

Circular Dichroism (CD) spectroscopy, nondenaturing Polyacrylamide Gel Electrophoresis (PAGE), and other techniques have been applied in the study of G4-DNA conformational states^40^,^41^,^42^,^43^,^44^,^45^. In this work, nondenaturing PAGE and CD spectra experiments were carried out to investigate the conformational structures of kanamycin-binding DNA oligonucleotides in the presence of 10 mM NaCl.

**Figure 1:**
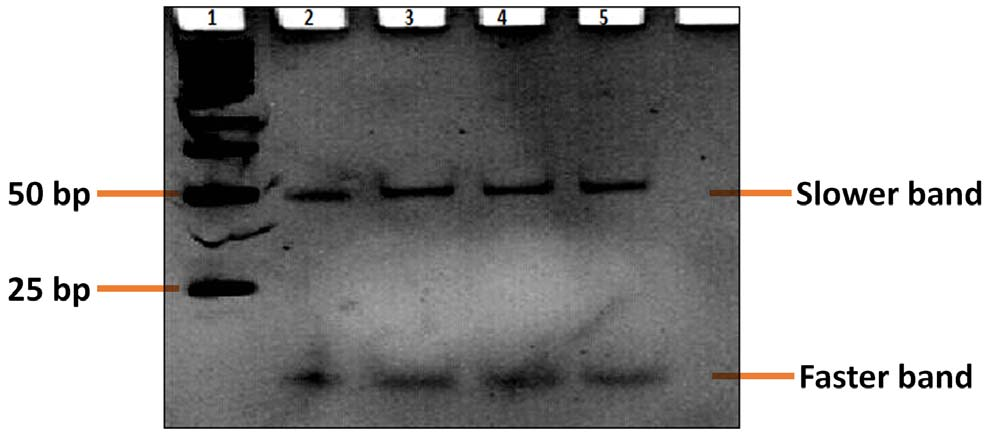
Nondenaturing 10% PAGE image of the kanamycin-binding aptamer (5′-TGG GGG TTG AGG CTA AGC CGA-3′) in the 10 mM Tris–HCl buffer (pH 7.2) containing 10 mM NaCl at 4°C (pH 7.2). (Lane 1, Marker; from Lane 2 to Lane 5: four parallel DNA samples at strand concentration of 2 μM).

Fig. 1 shows the results of nondenaturing PAGE for kanamycin aptamer (5′-TGG GGG TTG AGG CTA AGC CGA-3′) in 10 mM Tris–HCl buffer containing 10 mM NaCl (pH 7.2) at 4°C. We observe two distinct bands with clearly different mobilities for the DNA aptamer used, indicating that different conformational states have been formed for the guanine (G)-rich repetitive sequences^40^,^41^,^42^,^43^. The slower band indicates that it corresponds to an intermolecular G-quadruplex^42^ because an intramolecular G-quadruplex or a single-stranded DNA with 21 bases migrates faster than the 50-base pair duplex of 100 bases. As stated by Simonsson^46^, strand stoichiometry variation allows G-quadruplexes to be formed by the association of one, two, or four separate strands; however, three strand arrangements are conceivable but have yet to be substantiated. According to the proposed aptamer sequence (which is 21 bases in length) and the PAGE results, the intermolecular G4-DNA in our study should be formed by the association of four separate strands. On the contrary, a dramatic increase in the mobility of the faster band indicates that the band corresponds to an intramolecular G-quadruplex or a single-stranded DNA.

**Figure 2:**
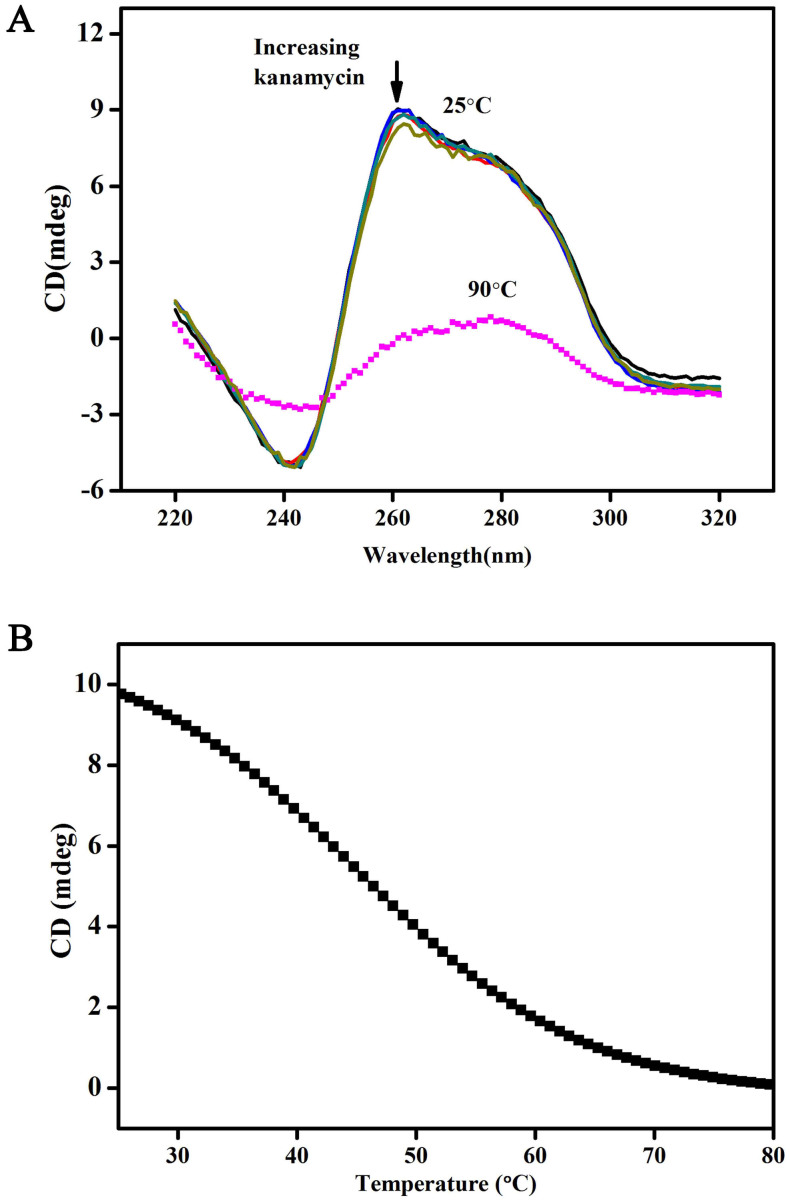
(A) CD spectra of the oligonucleotides at a strand concentration of 15 μM with kanamycin (Solid line, from top to bottom: 0, 1, 5, 10, 20, and 50 μM) at 25°C and without kanamycin (dotted line) at 90°C in the 10 mM Tris–HCl buffer containing 10 mM NaCl (pH 7.2); (B) CD melting curves for the oligonucleotides monitored at 260 nm in 10 mM Tris–HCl buffer containing 10 mM NaCl (pH 7.2).

To further verify the conformational state of the oligonucleotide, the CD spectra of the kanamycin-binding oligonucleotides are investigated and presented in Fig. 2. CD is remarkably sensitive to the conformational states of nucleic acids^44^. The CD spectra of the oligonucleotides at 25°C show a positive band at 265 nm and a negative band at 240 nm (Fig. 2A), which is the characteristic CD signature of a parallel G-quadruplex structure of DNA^42^,^43^,^47^. Notably, the dominant CD band at 265 nm is weakened at 90°C and shifted to the CD spectrum of an unstructured single strand^43^, indicating that G4-DNA is formed at 25°C. According to CD melting experimental results (Fig. 2B), the T_m_ value of the parallel G-quadruplexes in 10 mM NaCl solution is 49°C, suggesting that the oligonucleotides form a stabile parallel G-quadruplex under low-salt conditions. Results from both the nondenaturing PAGE and the CD spectra confirm that kanamycin-binding DNA aptamers can form stable G-quadruplex structures by themselves. Moreover, the G4-DNA is formed with parallel strand alignment. We have also investigated the effects of kanamycin on the CD spectra. As shown in Fig. 2A, almost no changes in the CD spectra were observed when different kanamycin concentrations (e.g. 1, 5, 10, 20, 50 μM) were spiked. The phenomenon, on the one hand, emphasizes the stability of the parallel G-quadruplex structures, which cannot be destroyed by the addition of kanamycin and, on the other hand, inspires us to design the turn-off biosensing strategy for kanamycin determination as follows.

### Principle of biosensing strategy

**Figure 3:**
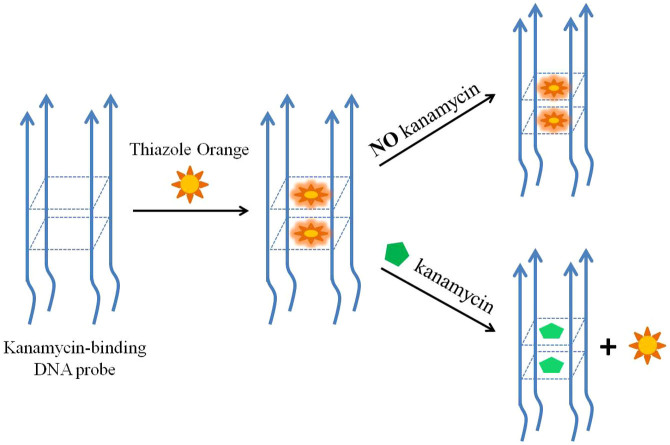
Schematic diagram of the sensing principle for kanamycin detection based on a G4-DNA aptamer-based FID assay.

As illustrated in Fig. 3, the principle of this proposed bioassay is based on the displacement of an “on–off” fluorescence probe of TO from the quadruplex matric by increasing amounts of kanamycin. Notably, a quadrimolecular G-quadruplex with parallel strand alignment is verified by the PAGE image and CD spectra. However, more experimental explorations and evidence are required to reveal the detailed molecular structure of the G4-DNA probe, which is beyond the scope of the current work. For the demonstration of biosensing strategy, the G4-DNA probe structure in Fig. 3 is hypothesized to share a guanine tetrad formed by the continuous guanines with the rest of the DNA strand left out like a tail. TO being virtually nonfluorescent when free in solution but strongly fluorescent when bound to quadruplex DNA^35^,^36^,^37^,^38^,^39^,^40^, the target-induced displacement leads to a decrease of the fluorescence that is inversely related to the kanamycin concentration. The detection principle is very similar to the G4-FID proposed by Teulade-Fichou's team in 2006^32^. However, previous studies are still limited in the range of determining the quadruplex- and duplex-DNA binding affinity and its selectivity for ligands^32^,^35^,^36^,^37^,^38^,^39^,^40^. In this work, we were inspired by the parallel G-quadruplex structure of kanamycin-binding DNA aptamers and developed the G4-DNA aptamer-based FID assay for the analysis application of label-free kanamycin quantification.

**Figure 4:**
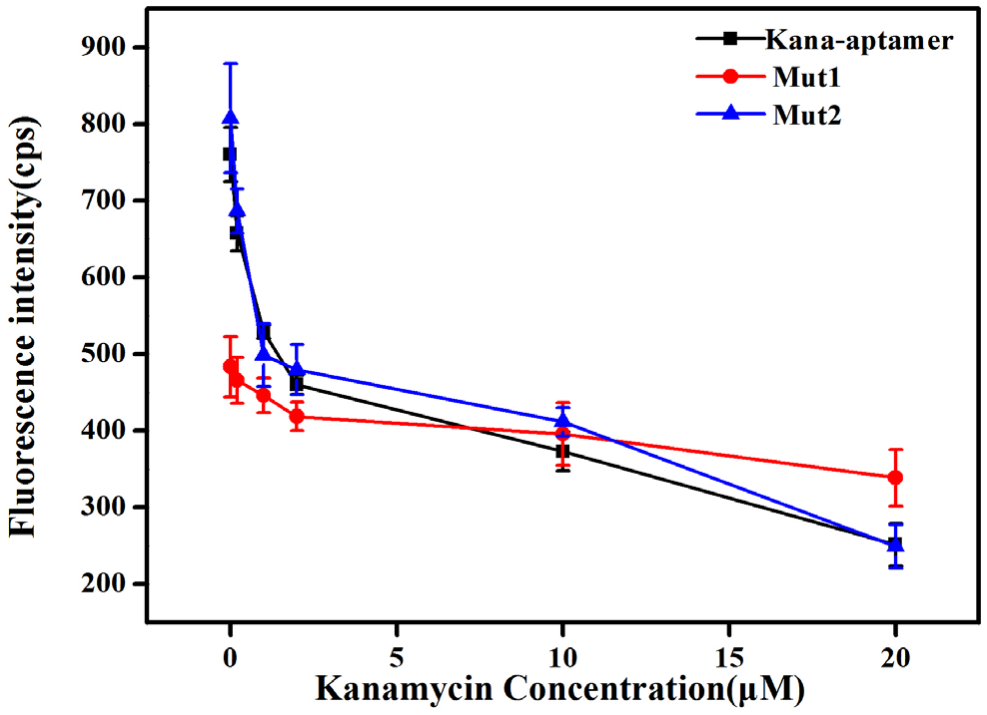
Fluorescence intensities at 530 nm and their corresponding kanamycin concentrations in 10 mM Tris–HCl buffer (pH 7.2) containing 10 mM NaCl by using kanamycin aptamer and its two mutant sequences. Error bars represent the standard deviation of triplicate measurements.

To provide support for the binding location of the G-quartet plane, two mutant sequences (Mut1 and Mut2) were investigated and their responses compared with the aptamer sequence towards kanamycin are shown in Fig. 4. The fluorescence intensity of TO was much lower when the Mut1 sequence with G-base mutants in the middle was used; however, the Mut2 sequence with G-base mutants on the 5′-end displays a comparable binding affinity towards kanamycin relative to the original aptamer. This suggests that kanamycin interacts significantly with kanamycin aptamer through binding to a two-layer guanine tetrad formed by the continuous guanines (GG region) in the middle of the sequence. We further envisage that the fluorescence enhancement of TO with kanamycin aptamer is due to intercalation of TO into the two-layer guanine tetrad structure as presented in Fig. 3.

Fig. 2A also further verifies that the fluorescence signal change caused by the addition of kanamycin is a displacement process. The addition of kanamycin does not perturb the G-quadruplex structure of kanamycin-binding DNA aptamer, as proven by the CD spectra even when the kanamycin concentration is threefold that of the DNA strand.

### Optimization of bioassay conditions

**Figure 5:**
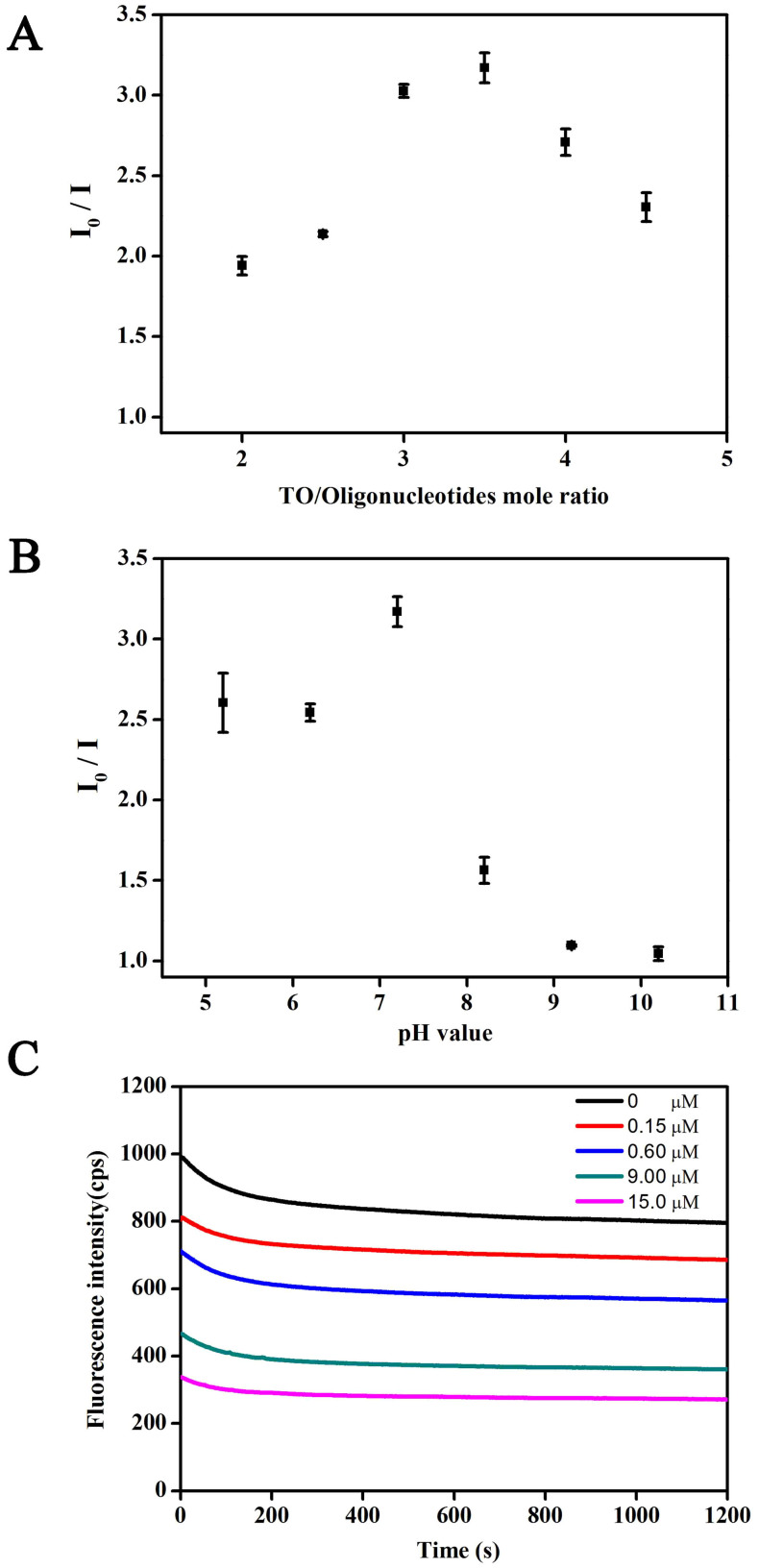
Signal ratios of fluorescence intensities (I_0_/I) before and after the addition of kanamycin to a final concentration of 20 μM at (A) different TO/oligonucleotide mole ratios by varying TO concentrations ranging from 20, 25, 30, 35, 40, and 45 μM with a fixed oligonucleotide strand concentration of 10 μM; and (B) different pH values ranging from 5.2, 6.2, 7.2, 8.2, 9.2, to 10.2. (C) Time course of fluorescence signals upon the addition of kanamycin at different concentrations (0, 0.15, 0.60, 9.00, and 15.00 μM) under the optimized pH of 7.2 and TO/oligonucleotide mole ratio of 0.35 μM to 10 μM. All measurements were conducted in 10 mM Tris–HCl buffer (pH 7.2) containing 10 mM NaCl. Error bars in A and B represent the standard deviations in three individual experiments.

Dye/DNA probe mole ratio, pH value, and reaction time are very critical factors for label-free aptamer-based bioassays based on the staining of the dye^3^,^29^,^30^. Fig. 5A shows the effect of TO/oligonucleotide mole ratio on the ratios of fluorescence intensities (I_0_/I) in the absence and presence of kanamycin to a final concentration of 20 μM. I_0_/I is a very sensitive index for the optimization of detection conditions to gain the highest signal-to-noise ratio^3^,^29^,^30^. I_0_/I is found to reach the maximum when the mole ratio is 3.5, indicating that the optimized TO concentration is 35 µM at the fixed G-quadruplex concentration of 10 µM. Higher or lower ratio values could damage the relative fluorescence intensities of the system. We have also observed the effect of buffer pH value on the I_0_/I value for kanamycin detection as shown in Fig. 5B. The maximum value of I_0_/I appeared at the pH value of 7.2; therefore, pH 7.2 was adopted in the subsequent experiments. Moreover, the alkaline conditions sharply decrease the relative fluorescence intensities of the system, which is in accord with previously reported studies^48^. Fig. 5C depicts the time course of fluorescence signals of the sensing system upon the addition of varying concentrations of kanamycin under the optimized pH value of 7.2 and TO/oligonucleotide mole ratio of 35 μM to 10 μM. The fluorescence intensity is found to decrease as soon as kanamycin is added. The fluorescence signal gradually decreases, and then reaches a state of relative equilibrium within 10 min, which is suitable for the target quantification. Results not only indicate that the TO is a very suitable dye for staining the G-quadruplex structure of DNA aptamer but also reveal that the displacement of TO in the presence of kanamycin is a rapid process. Also, the fluorescence intensity values decrease accordingly with the increased kanamycin concentrations, which is in accord with our speculation toward the proposed biosensing strategy.

### Detection performance for kanamycin

#### Sensitivity

**Figure 6:**
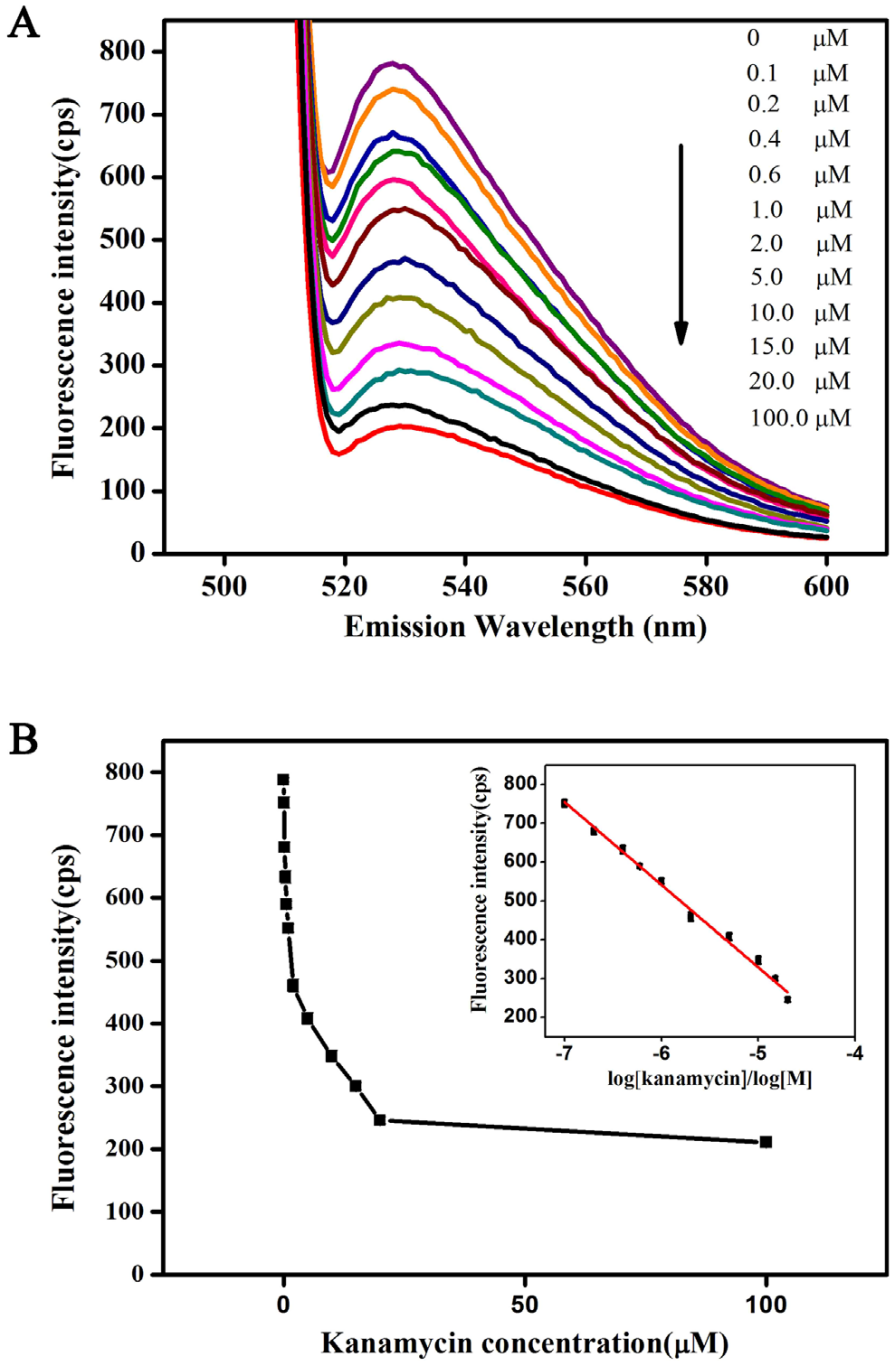
(A) Typical fluorescence spectra of TO/Kanamycin-binding DNA aptamer in the absence and presence of different kanamycin concentrations ranging from 0.1, 0.2, 0.4, 0.6, 1, 2, 5, 10, 15, 20, and 100 μM in 10 mM Tris–HCl buffer (pH 7.2) containing 10 mM NaCl. (B) Relationship between the fluorescence intensities at 530 nm and their corresponding kanamycin concentrations. Inset: calibration plot for kanamycin detection (error bars represent the standard deviations in three individual experiments).

Under the optimized detection conditions, Fig. 6A shows the fluorescence spectra of TO/Kanamycin-binding DNA aptamer in the absence and presence of different kanamycin concentrations ranging from 0.1, 0.2, 0.4, 0.6, 1, 2, 5, 10, 15, 20, and 100 μM. Encouragingly, the fluorescence intensity of TO at the maximum emission wavelength of 530 nm is weakened continually with increasing concentrations of kanamycin because of the displacement of TO from G-quadruplexes aptamer by kanamycin. A maximum fourfold decrease in the emission response of the system has been observed in the presence of 100 μM of kanamycin, indicating high sensitivity.

The relationship between fluorescence intensities at 530 nm and their corresponding kanamycin concentrations is depicted in Fig. 6B. The inset in Fig. 6B shows the calibration plot for kanamycin detection. According to the above results, the detection limit of this bioassay is calculated to be 59 nM using the three-sigma method^3^, which is comparable or more sensitive than the previously reported luminescent (143 nM) and electrochemical (2000 nM) assays^3^,^49^, and slightly less sensitive to colorimetric (1 nM and 25 nM)^2^,^50^ and gold nanocomposite (9.4 nM)^15^ assays. The linear range of the proposed label-free bioassay is from 0.1 μM to 20 μM.

#### Selectivity

**Figure 7:**
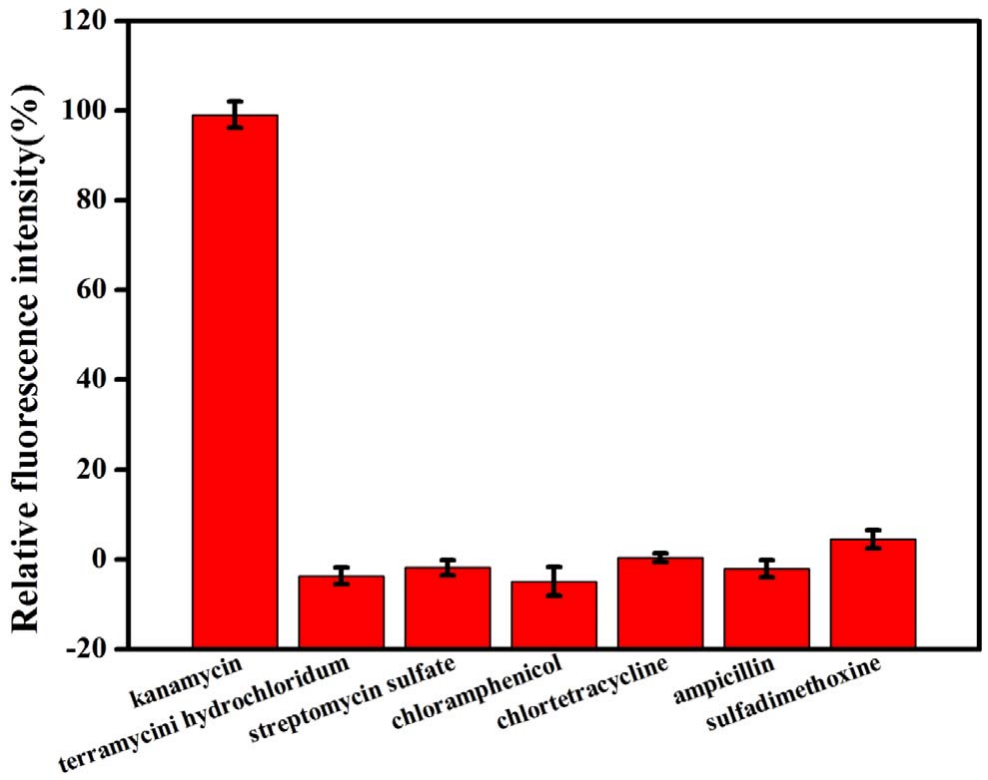
Relative fluorescence change of TO and G4-DNA complex (mole ratio of 3.5) at 530 nm upon the addition of (1) kanamycin, (2) terramycin hydrochloride, (3) streptomycin sulfate, (4) chloramphenicol, (5) chlortetracycline (6) ampicillin and (7) sulfadimethoxine (all at a final concentration of 20 μM) in 10 mM Tris–HCl buffer (pH 7.2) containing 10 mM NaCl. Error bars represent the standard deviation in three individual experiments.

To further validate the selectivity of this proposed bioassay for kanamycin, the responses of the system to six other antibiotics including terramycin hydrochloridum, streptomycin sulfate, chloramphenicol, chlortetracycline, ampicillin and sulfadimethoxine are investigated. At 20 µM of analytes, only kanamycin can cause significant fluorescence intensity in the sensing system as shown in Fig. 7. The addition of other antibiotics results in only slight increases (<5%) in the fluorescent enhancement. The results demonstrate that our assay is highly selective toward kanamycin over other classes of antibiotics. To further investigate the effect of analogous antibiotics on the fluorescence response of the bioassay, a competition study was carried out. As we expected, no significant differences in the maximal fluorescence response (%) of the proposed bioassay to kanamycin were found in the presence of the same concentration of interfering antibiotics compared with the absence of interfering antibiotics in the system (Fig. S1).

#### Recovery

In order to validate the reliability of the proposed method, a bioassay is applied for the detection of kanamycin in milk samples. Under the optimized detection conditions and samples pretreatment as shown in Methods, the fluorescence signals were almost the same as those responding to the blank buffer solutions before the dairy samples were spiked with kanamycin. And then, the spiked concentrations at three levels of 0.15, 1.0, and 10.0 µM were measured and compared with the added values and the results are summarized in Table 1. The average recoveries are found to vary from 80.1% to 98.0%, demonstrating the satisfactory accuracy of the developed aptamer-based label-free bioassay.

## Discussion

The kanamycin-binding DNA aptamer (5′-TGG GGG TTG AGG CTA AGC CGA-3′ or 5′-TGG GGG TTG AGG CTA AGC CGA C-3′) has been reported for a few years and used for the construction of aptamer-based kanamycin biosensing technologies^2^,^3^,^15^,^16^. Structural characterization of DNA aptamers and its binding principle toward kanamycin still lacks informative investigation and accessible opinions. Although, Leung et al. speculate that the kanamycin aptamer changes from a random-coiled structure into a hairpin-like structure upon the addition of kanamycin, which facilitates the intercalation of the luminescent platinum (II) complex probe, resulting in an enhanced luminescence signal^3^. However, the turn-on reaction could not be reproduced in our studies when we used the famous nucleic acid dye of SYBR Green I^29^,^30^ instead of platinum(II) complex probe. Therefore, we tried to address the issue by CD spectroscopy and nondenaturing PAGE^40^,^41^,^42^,^43^,^44^,^45^ to study the DNA conformational state of kanamycin aptamers. Both methods are remarkably sensitive to the conformational states of nucleic acids, especially the CD spectroscopy^44^. Benefitting from these help, we first verify that the kanamycin DNA aptamer can form stable parallel G-quadruplex structures by themselves (Figs. 1 and 2). The addition of kanamycin also has a negligible effect on the conformational state of kanamycin DNA aptamer (Fig. 2). In fact, Arachchilage et al. have reported a G-quadruplex RNA aptamer for kanamycin recognition^51^, but RNA aptamers have no advantage over DNA in the design of bioassays because of their instability.

The abovementioned experimental phenomena also inspired us to develop a novel label-free strategy for the detection of kanamycin based on the staining of nucleic acid dyes. Label-free detection is always very attractive because of its remarkable advantages such as cost-effectiveness and simplicity, which can also be highly sensitive and selective through the use of fluorescent nucleic acid dye, such as SYBR Green I and TO. SYBR Green I is known for its high sensitivity^29^,^30^,^49^ and TO is commonly used for the quadruplex- and duplex-DNA stain in the G4-FID assays^32^,^33^,^34^,^35^,^36^,^37^,^38^. In this work, the critical success factor in developing a label-free G4-DNA aptamer-based bioassay for kanamycin is attributed to the choice of workable nucleic acid dye. We began with the SYBR Green I as the fluorescent probe because it is widely used. As shown in Fig. S2 for the time courses of the two sensing systems using SYBR Green I and TO, respectively, upon the addition of varying concentrations of kanamycin under the optimized detection condition, the fluorescence intensities of SYBR Green I probe continued to decline via the detection time, with the result that no relative equilibrium state could be reached compared with using TO. This indicated that SYBR Green I was not suitable for the staining of G4-DNA because the instability of fluorescence intensities ruins the bioassay for quantification. Instead, TO is promising in the development of other G4-DNA-based label-free fluorescence bioassays.

In our proposed strategy, by taking TO as the fluorescent probe, TO becomes strongly fluorescent upon binding to kanamycin G4-DNA aptamers. However, the addition of kanamycin causes the displacement of TO from the G4-DNA–TO conjugate, thereby resulting in a decrease in the fluorescence signal inversely related with the kanamycin concentration. In the buffer solutions, the detection limit of the proposed assay decreased to 59 nM with a linear working range of 0.1 μM to 20 μM for kanamycin, which is impressive compared with other reported studies^2^,^3^,^15^,^49^,^50^. A good selectivity of this bioassay for kanamycin over six other antibiotics is also proven. The recovery study confirms the application potential of the proposed bioassay in the measurement of kanamycin in milk samples.

In a word, the established G4-DNA aptamer-based FID assay method shows highly sensitive, selective, cost-effective, rapid and satisfactory recovery for kanamycin detection. The method also paves the way for kanamycin biosensing in applications such as food safety monitoring, water quality monitoring, and chemical production. This work also provides a good reference for the development of similar fluorescent G4-DNA-based bioassays in the future.

## Methods

### Reagents and materials

Kanamycin, terramycin hydrochloridum, streptomycin sulfate, chloramphenicol, chlortetracycline, ampicillin, and sulfadimethoxine were purchased from Beijing Songyuan Co., Ltd. (Beijing, China) and TO (10 mM dissolved in DMOS buffer as the stock solution) was from Sigma–Aldrich (USA). Other chemicals used as buffers and solvents were purchased from Biodee Co., Ltd. (Beijing, China). All chemicals were of analytical grade if not specified and were used as received without further purification.

Kanamycin-binding DNA oligonucleotides (5′-TGG GGG TTG AGG CTA AGC CGA-3′), as reported by previous studies^2^,^3^, and two mutant aptamer sequences (Mut1: 5′-TGG GGG TTG AAA CTA AGC CGA-3′ and Mut2: 5′-TGG AAA TTG AGG CTA AGC CGA-3′) (base mutants underlined) were all synthetically produced by Sangon Biotechnology Co., Ltd. (Shanghai, China) and pretreated as follows. The DNA oligonucleotide stock solution was prepared by diluting the kanamycin-binding oligonucleotides to a strand concentration of 10 μM using 40 mM Tris–HCl buffer (pH 7.2) containing 100 mM NaCl. The stock solution was heated at 95°C for 5 min and cooled to room temperature over a 90-min period to favor the G-quadruplex formation. The mutant aptamer sequences were pretreated in the same way before using. In order to acquire enhanced signals, another concentrated oligonucleotide stock solution was prepared at 100 μM according to the abovementioned procedures, specifically for the CD spectroscopy. 10 mM kanamycin stock solution was prepared with deionized water. All stock solutions were stored at 4°C before use.

All buffers were prepared either in DEPC-treated deionized water or biomolecule grade deionized water (RNA Nuclease and DNA Nuclease free), noted hereafter as DI water.

### Nondenaturing PAGE

Kanamycin aptamer samples (10 μL of 0.5 μM) were separated by nondenaturing PAGE on 10% acrylamide (19:1 acrylamide/bisacrylamide) gels at 5 V cm^−1^ and 4°C. Gels were stained with GelRed Acid Gel Stain (Biotium, US) and imaged under UV illumination using FLS-5100 film (Fuji Photo Film Co., Ltd., Tokyo, Japan). All measurements were carried out in 10 mM Tris–HCl buffer (pH 7.2) containing 10 mM NaCl.

### CD Spectroscopy

The CD spectra of Kanamycin-binding DNA oligonucleotides were measured by a Chirascan™ circular dichroism spectrometer (Applied Photophysics, UK). A quartz cuvette with a 1-mm path length was used for the spectra recorded over wavelengths ranging from 220 nm to 320 nm at 1 nm bandwidth, 1 nm step size, and 0.5 s per point. The 15 μM strand concentration of oligonucleotides was prepared by diluting the concentrated stock solution with 10 mM Tris–HCl buffer (pH 7.2) containing 10 mM NaCl and pretreated as mentioned previously. Buffer blank corrections were made for all spectra.

### Fluorescence measurement for kanamycin detection

Kanamycin stock solution was diluted as a series of kanamycin concentrations (5, 10, 20, 30, 50, 100, 250, 500, 750 µM, and 1 mM, and 5 mM) with DI water. The stock solution of DNA oligonucleotides was diluted with 10 mM Tris–HCl buffer (pH 7.2) containing 10 mM NaCl to be a strand concentration of 10 μM. A series of TO solutions was prepared with DI water.

The optimized detection procedures for kanamycin are shown in the following steps: 10 μL of 10 μM oligonucleotides, 10 μL of 35 μM TO and 20 μL of kanamycin solution at different concentrations were successively added into 960 μL of 10 mM Tris–HCl buffer (pH 7.2) containing 10 mM NaCl. The mixture was vortexed for 10 min before fluorescence measurements. The final concentrations of kanamycin in the sensing system were, respectively, 0.1, 0.2, 0.4, 0.6, 1, 2, 5, 10, 15, 20, and 100 µM. If not specified, all analyte concentrations mentioned in this work refer to the final concentration of kanamycin in the samples for fluorescence measurements. The fluorescence spectra were recorded using a Hitachi F-7000 fluorescence spectrophotometer (Hitachi, Japan). Excitation and emission wavelengths were 501 and 530 nm, respectively. All measurements were conducted at room temperature (25°C).

To provide support for the proposed binding mechanism, two mutant aptamer sequences (Mut1 and Mut2) instead of the kanamycin-binding aptamer, respectively, were used to investigate their responses towards kanamycin according to the abovementioned procedures.

### Optimization of bioassay conditions

Before confirming the optimized detection procedures mentioned previously, we also investigated the effects of key detection conditions as other reported label-free, aptamer-based bioassays based on the staining of dye^3^,^29^,^30^, including the TO/oligonucleotide mole ratio, the buffer pH values, and reaction time under vortex in order to optimize the fluorescence response of the system for kanamycin detection. The TO/oligonucleotide mole ratios were optimized by fixing the oligonucleotides at 10 μM and varying TO concentrations at 20, 25, 30, 35, 40, and 45 μM. The buffer pH values ranged from 5.2, 6.2, 7.2, 8.2, 9.2, to 10.2, respectively. The ratios of fluorescence intensities (I_0_/I) were recorded and used as the optimization criteria. I_0_ and I were the fluorescence intensities in the absence and presence of kanamycin to a final concentration of 20 μM. We also observed the time course of fluorescence signals when the mixture was freshly prepared under the optimized pH value and TO/oligonucleotide mole ratio in order to fix the reaction time under vortex. Notably, the other detection conditions not mentioned were the values used in the optimized detection procedures. All measurements were conducted at room temperature.

### Selectivity and recovery in milk samples

In the selectivity experiment, six other antibiotics, terramycin hydrochloridum, streptomycin sulfate, chloramphenicol, chlortetracycline, ampicillin and sulfadimethoxine (all at 20 µM) were, respectively, chosen instead of kanamycin. In a further step, we chose a mixed kanamycin sample (20 µM kanamycin mixed with terramycin hydrochloridum, streptomycin sulfate, chloramphenicol, chlortetracycline, ampicillin, and sulfadimethoxine, each at 20 µM) for evaluation.

The liquid dairy milk was bought from the local supermarket. liquid milk (2 mL), acetonitrile (3 mL), and 10% trichloroacetic acid (15 mL) were added into a centrifuge tube and vortexed for 1 min to precipitate protein and dissolve organic substances. The mixture was further centrifuged at 12,000 rpm for 5 min to enhance the separation performance. Subsequently, the middle liquid layer was spiked with different kanamycin solutions to final concentrations at three levels (0.15, 1.0, and 10.0 μM). Detection procedures were carried out according to the abovementioned optimized conditions for kanamycin detection.

## Author Contributions

Y.-P.X. and X.-H.Z. designed and performed all the experiments, and wrote the manuscript. C.L. and H.-C.S. discussed the results and commented on the manuscript. All the authors reviewed the manuscript.

## Supplementary Material

Supplementary Information Supplementary Material for Label-free detection of kanamycin based on a G-quadruplex DNA aptamer-based fluorescent intercalator displacement assay

## Figures and Tables

**Table 1:** Recovery of kanamycin in milk samples using the proposed bioassay

	Kanamycin (μM)		
Sample	Spiked	Found mean^a^ ± SD^b^	Recovery (%)
Milk 1	0.15	0.147 ± 0.010	98.0
Milk 2	1.0	0.801 ± 0.011	80.1
Milk 3	10.0	9.154 ± 0.646	91.5

^a^Mean value of three individual determinations.

^b^Standard deviation.
