# Supplementary material for: Label-free detection of kanamycin based on a G-quadruplex DNA aptamer-based fluorescent intercalator displacement assay
Source: Sci Rep. 2015 Jan 30;5:8125. doi: 10.1038/srep08125 (PMC4311242; doi:10.1038/srep08125)
Supplement: Supplementary Information — Supplementary Material for Label-free detection of kanamycin based on a G-quadruplex DNA aptamer-based fluorescent intercalator displacement assay [file srep08125-s1.doc]

**Supplementary Material**

***for***

**Label-free detection of kanamycin based on a G-quadruplex DNA aptamer-based fluorescent intercalator displacement assay**

Yun-Peng Xing^a.b^, Chun Liu^b^, Xiao-Hong Zhou^*a^, Han-Chang Shi^a^

a. State Key Joint Laboratory of ESPC, School of Environment, Tsinghua University, Beijing 10084, China

b. School of Environmental Science and Engineering, Hebei University of Science and Technology, Shijiazhuang 050000, China

Corresponding author: [xhzhou@mail.tsinghua.edu.cn](mailto:xhzhou@mail.tsinghua.edu.cn)

**Fig. S1** Relative fluorescence change of TO and G4-DNA complex (mole ratio of 3.5) at 530 nm upon the addition of (1) 20 μM kanamycin and (2) 20 μM kanamycin + 20 μM terramycin hydrochloridum, (3) 20 μM kanamycin + 20 μM streptomycin sulfate, (4) 20 μM kanamycin + 20 μM chloramphenicol, (5) 20 μM kanamycin + 20 μM chlortetracycline, (6) 20 μM kanamycin + 20 μM ampicillin and (7) 20 μM kanamycin + 20 μM sulfadimethoxine in 10 mM Tris–HCl buffer (pH 7.2) containing 10 mM NaCl. Error bars represent the standard deviations in three individual experiments.

**Fig. S2** Time course of fluorescence signals in two sensing systems using SYBR Green I and TO as the fluorescence probe, respectively, upon the addition of kanamycin at different concentrations (0, 0.60, and 15.00 μM) under the optimized detection conditions.
